# Use of Graphene and Its Derivatives for the Detection of Dengue Virus

**DOI:** 10.3390/bios13030349

**Published:** 2023-03-06

**Authors:** Reshmi Dutta, Kokilavani Rajendran, Saikat Kumar Jana, Lilly M. Saleena, Suvankar Ghorai

**Affiliations:** 1 Department of Biotechnology, SRM Institute of Science and Technology, College of Engineering and Technology, SRM Nagar, Kattankulathur, Kanchipuram, Chennai 603203, India; 2 Department of Biotechnology, National Institute of Technology, Arunachal Pradesh 791109, India; 3 Department of Microbiology, Raiganj University, Raiganj 733134, India

**Keywords:** graphene, diagnostics, graphene oxide, reduced graphene oxide, dengue virus, point of care, sensors

## Abstract

Every year, the dengue virus and its principal mosquito vector, *Aedes* sp., have caused massive outbreaks, primarily in equatorial countries. The pre-existing techniques available for dengue detection are expensive and require trained personnel. Graphene and its derivatives have remarkable properties of electrical and thermal conductivity, and are flexible, light, and biocompatible, making them ideal platforms for biosensor development. The incorporation of these materials, along with appropriate nanomaterials, improves the quality of detection methods. Graphene can help overcome the difficulties associated with conventional techniques. In this review, we have given comprehensive details on current graphene-based diagnostics for dengue virus detection. We have also discussed state-of-the-art biosensing technologies and evaluated the advantages and disadvantages of the same.

## 1. Introduction

Dengue virus (DENV), a member of the family Flaviviridae, is a single-stranded positive-sense RNA virus with a genome of 11 kb [1] and is transmitted by the female *Aedes aegyptii* mosquito vector [2]. DENV consists of three structural proteins, namely the membrane-associated protein M, core protein C, and envelope protein E, which are responsible for shaping the structure of the virus. In addition, it contains seven non-structural proteins, NS1, NS2A, NS2B, NS3, NS4A, NS4B, and NS5, which play a role in the replication of the virus and many more cellular functions. The different serotypes of dengue are DENV-1, DENV-2, DENV-3, and DENV-4. Infection caused due to any of these serotypes is usually asymptomatic or flu-like symptoms develop, which might lead to severe dengue hemorrhagic fever or dengue shock syndrome [1]. A global study showed that about 390 million people get infected by dengue annually, of which 96 million exhibit clinical manifestations [2]. As of 23 September 2022, 110,473 dengue cases have been reported in India [3].

Conventional methods for dengue detection are laborious, time-consuming, costly and require skilled professionals. Hence, nanomaterial-based sensors can be a good option for dengue detection as they surpass all the issues mentioned above that conventional methods face. Nanomaterials have exceptional qualities, making them an ideal platform for usage in biosensors. They can be used for certain purposes by regulating physical and chemical properties, namely size, morphology, surface charge and solubility [4]. Such properties make nanomaterials ideal for their utility in biosensors to enhance target-specific reactions which respond to biochemical reactions such as temperature, pH, and the existence of enzymes [5]. Nanomaterials might boost the sensitivity of present diagnostic techniques due to features like high reactivity, adsorption capacity, particle size, and physicochemical bonding capacity. Utilizing nanoparticles might lead to enhancements in technology regarding time, specificity, mobility and convenience [4].

**Figure 1:**
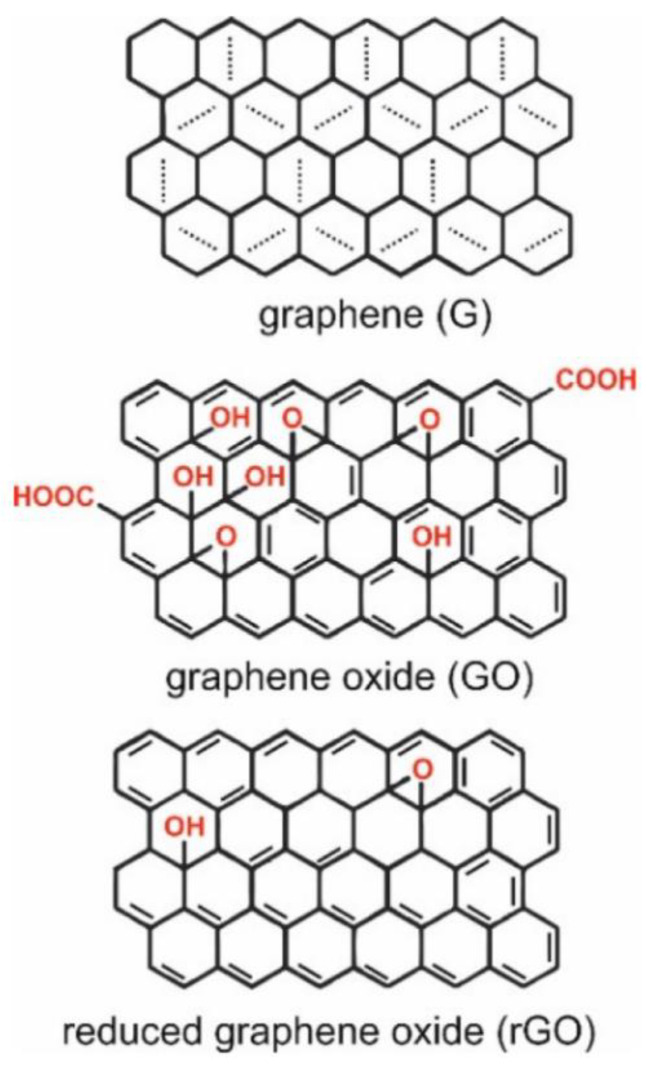
Structures of graphene (G), graphene oxide (GO), and reduced graphene oxide (rGO) (Reused with permission from Nanomaterials, MDPI [16]). Aromaticity in graphene is local, with two π-electrons located over every hexagon ring [17].

Graphene is one such material which is being used extensively in the development of sensors. Graphene is a premium material formed by a single-thick layer of sp^2^ hybridized carbon atoms arranged in a honeycomb lattice [6]. It is an extremely thin material and occurs as a few layers of graphite [7]. It displays outstanding electrochemical properties such as high thermal conductivity (above 3000 W m K^−1^) and low redox potential (−0.5 V–1.2) [8]. Optical absorption in the infrared region, a high surface area (2630 m^2^/g), completely impermeable to gas [9], and mechanical strength (about 1100 GPa) [10], are some of the properties for which graphene is used for diagnostic purposes [9,10]. The different forms of graphene include graphene oxide (GO), reduced graphene oxide (rGO), graphene sheets [7], and layered graphenes such as a few layered graphenes and multilayered graphene [7,11]. Some of the widely studied derivatives of graphene are GO and rGO (Figure 1). They display properties similar to graphene, such as flexibility, transparency, and low cytotoxicity. The hydrophilic nature and presence of reactive functional groups make them an ideal material to be used in a biosensor [12]. The hydrophilic feature plays a key role in assembling the biosensor as it permits film preparation by drop casting, spin coating, ink-jet printing, and electrode material processing. Research on these materials has successfully led to their use in different fields such as electronics, optics, sensors, and filtration [8]. The synthesis of GO involves vigorous oxidation of graphite–graphene solutions and hence leads to the production of a complex material having a variety of functional groups whose properties and quantity rely on the method of preparation. There is a tendency for functional groups of GO to get modified on the surface due to the attachment of redox species such as enzymes, peptides, or DNA and RNA [13]. In an acidic medium, graphite is oxidized to GO using Hummers’ and Offemans’ methods [14]. GO’s disrupted sp^2^ hybridized bonding is responsible for its electrically insulating nature, however, GO can also be a good semiconductor when oxidized extensively [15].

When graphene is converted to GO, it increases the hydrophilicity of the surface and results in the formation of big functional groups [18]. The presence of these oxygenated groups (hydroxyl and epoxy) thus aids in the formation of a stable dispersion in aqueous media and other polar solvents [14] and further enables biochemical and bioconjugation reactions to occur with ease [14,19]. GO is a remarkable adsorbant of proteins and antibodies, making it a useful biomaterial [20]. GO, on reduction, produces rGO by removing the oxidized functional groups of GO [21]. The reduction procedure establishes considerable changes involving surface properties in rGO [20]. The absence of most of the oxygen-containing functional groups from GO partly restores the sp^2^ structure [22] and improves the rGO conductivity [22,23]. Some unique properties of rGO involve conductivity, fluorescence quenching, peroxide-like enzyme activity, intrinsic Raman activity, oxidation-reduction capacity, and the ability to anchor different nanoparticles, enzymes, proteins, and nucleic acids [13]. While GO has inferior electrical properties, it is a better conductor and can retain its ability to disperse in water [24]. GO and rGO can be utilized in the preparation of a wide range of graphene-based nanocomposites. Some materials incorporated with graphene derivatives are nanoparticles, quantum dots, nanoclusters, polymers, and a variety of biomolecules [25]. Graphene and its derivatives undergo non-covalent interactions such as London forces, polarization, hydrophobic effects, and electrostatic force of attraction with adsorbates [26]. Such interactions confirm the adsorbate’s contact with a surface, leading to alterations in electronic characteristics that could be used in sensing. GO possesses a high loading ratio for biomolecules which can go up to 200% compared to other nanocarriers. This could be a useful feature for the development of sensitive electrochemical immunosensors [27]. A noteworthy point could be that 2D functionalized graphene derivatives and composites might undergo restacking. Restacking happens when multilayer Van der Waals materials are formed due to the powerful interaction of non-covalent interlayers. Developing a firm 3D network is crucial for yielding new and stable graphene nanocomposites that do not undergo restacking and possess beneficial features such as high surface area, tunable electronic features, improved conductivity and electrocatalytic characteristics, available inner space, and improved mechanical stability. Such features are suitable for systematic and selective biosensing [28].

Graphene and its derivatives offer considerable merits in biosensing applications for pathogenic virus detection. These materials are more conductive electrically and thermally, flexible, not heavy, biocompatible, and have a large surface area which is a gold standard for electrochemical platforms. Graphene, GO, and rGO successfully quench photoluminescence. Incorporating befitting nanomaterials into graphene and its derivatives can improve the quality of the known detection methods, e.g., Au–Ag nanoparticles transmit a notable surface-enhanced Raman scattering (SERS) signal. The sensor’s sensitivity is related to the transforming electronic features of graphene when the virus adsorbates are present. The large surface area of graphene-based materials helps the adsorption of analytes from the environment. When antibodies, aptamers, or nucleic acids are incorporated into graphene and its derivatives, they can bind to counterparts specific to a particular virus. This enhances the selectivity of a biosensor [13]. Therefore, biosensors utilizing graphene and its derivatives can be used efficiently for the detection of not only the dengue virus but also other upcoming pathogenic viruses.

This manuscript focuses on the different diagnostic methods involving graphene for dengue detection, which might help researchers working with graphene for developing sensors.

## 2. Extensive Use of Graphene in the Detection of Viruses

The light, chemically stable, and conductive nature of graphene makes it a success in its use in the detection of viruses. The incorporation of functional groups into the hybrid structure leads to swift virus detection [13]. Graphene and its derivatives are often used in the detection of viruses due to their high specificity and less time expenditure. Polymerase chain reaction (PCR) and loop-mediated isothermal amplification (LAMP) are thought to be sensitive techniques in the detection of the foot and mouth disease virus (FMDV) [29]. PCR has certain limitations, which lead to the generation of false positive results. A nano-PCR system, which uses graphene oxide–gold nanoparticles nanocomposites (GO-AuNPs), exhibits improved PCR efficiency [30].

In recent years rGO has found wide application in electrochemical biosensing for the detection of the H1N1 (haemagglutinin type 1 and neuraminidase Type 1) strain of the influenza virus. Label-free detection of the H1N1 virus was done using an electrochemical immunosensor coated with rGO and incorporated with a microfluidic platform [31]. The rGO obtained from shellac, used in label-free electrochemical biosensors, proved to be a cost-effective method for the detection of the influenza virus H1N1 [32].

Low-cost ultrasensitive biosensors using graphene and its derivatives exhibit excellent and promising results in the detection of human papilloma virus (HPV16) DNA in patients suffering from HPV16-positive head and neck cancer [33]. Reduced graphene oxide field effect transistors (rGO-FETs) are used for highly sensitive and selective detection of HPV16 E7 protein. To acquire selective sensing, specific probes are used on the conducting rGO channel of the transistor [34].

Sulfonated magnetic nanoparticles functionalized with reduced graphene oxide (SMRGO), having antiviral properties, were used to eliminate herpes simplex virus type 1 (HSV-1). Graphene has remarkable photothermal properties that inactivate the viruses captured with near-infrared (NIR) irradiation [35]. Graphene-mediated surface-enhanced Raman scattering (G-SERS) is often used to identify norovirus (NoV) in a sample of human faeces. In this unique biosensing system, magnetic derivative molybdenum trioxide nanocubes functioned as the SERS nanotag, and single-layer graphene oxide (SLGO) served as the signal reporter. With this technology, rapid signal amplification for the detection of NoV could be achieved [36].

Detection of the hepatitis C virus (HCV) could be made using a highly sensitive assay that involves the use of reduced graphene oxide nanosheets (rGONS) and a hybridization chain reaction (HCR) amplification technique. This method uses the selective adsorption capacity of rGONS to fluorophore probes with different conformations [37]. The enhanced fluorescence quenching capacity of rGO leads to inverse upregulation of the assay sensitivity by a reduction in the background signal of the probes [38,39,40]. The methods of detecting different viruses using graphene are listed in Table 1.

## 3. Conventional Methods Used for the Detection of the Dengue Virus

The conventional methods for the detection of dengue virus include serological tests; IgM-based tests, IgG-based tests, IgM/IgG ratio tests, haemagglutination inhibition tests, plaque reduction neutralization tests, NS1 tests; PCR-based tests, etc. Each diagnostic test differs in cost and time and requires highly developed medical facilities and trained technicians. Some graphene biosensor based assays provide a good alternative to conventional methods and are cost effective, rapid and give accurate results [41]. The current conventional methods of detecting dengue are listed in Table 2.

### 3.1. Serological Tests

They are commonly used for dengue detection due to their cost effectiveness and simple operation in comparison to molecular or cell-culture-based tests. Nagar et al. identified the specific peptide sequence of envelope E and NS1 proteins that depicted a worthwhile immunoreactivity with dengue-specific IgM and IgG antibodies in a patient’s serum. Certain peptides of the E protein and NS1 protein exhibited outstanding immunoreactivity, sensitivity, and specificity. Such peptides can be utilized in the development of diagnostic kits for the identification of the dengue virus [42]. The different serological tests are discussed below.

#### 3.1.1. IgM-Based Tests

An infection in the human body leads to the production of conventional antibodies such as IgG, IgM, IgA, and IgD. Symptoms usually appear in about five days, followed by the appearance of IgM antibodies [43]. IgM is produced first in the body, whereas IgG follows after a few days of IgM appearance. In the course of the secondary infection, IgG is produced first after the initiation of symptoms, whereas IgM antibodies are produced within a few days at low quantities [44]. An individual infected by dengue for the first time would have a higher IgM response, whereas later, for secondary infections, there would be more IgG antibody response [43]. An enzyme-linked immunosorbent assay (MAC ELISA) was used to detect IgM antibodies in dengue [45]. This technique exhibits a high sensitivity and specificity of 90% and 98% in samples taken following seroconversion [46]. However, the major shortcoming of all IgM antibody-based assays is a cross reaction with other viruses of Flaviviridae and might give improper results if patients were recently infected with DENV [47].

#### 3.1.2. IgG-Based Tests

IgG-based tests could be utilized to diagnose a present infection or an infection in the past. The IgG antibodies are produced in the body after IgM production, and they stay in the body for a prolonged period of time [48]. A fourfold rise in IgG antibodies can be correlated with a recent infection [49]. The principle involved here is that the antibodies which appear first following a primary infection, show low avidity to an antigen in comparison to the antibodies that are produced later on. This method is swift, simple, and appropriate for large-scale surveillance studies. In comparison to the haemagglutination inhibition assay, this method exhibits greater sensitivity. However, it faces cross reactivity with other Flaviviridae viruses and is also unable to determine the DENV serotype which causes the infection [47].

IgG tests could be more useful in some circumstances, however, NS1-ELISA and IgM-based tests are more effective in acute infections [50].

#### 3.1.3. IgM/IgG Ratio Tests

A first-occurrence infection and a later infection could be distinguished by an IgM/IgG ratio test [51]. Optical density values are obtained from the IgM ELISA and IgG ELISA tests. If the test value is higher than 1.32, it is estimated to be a primary infection, whereas a value lower than 1.32 is considered a secondary infection [52].

#### 3.1.4. Haemagglutination Inhibition Test (HI)

This is a definitive test for primary and subsequent dengue infections, but this test is unable to detect early infections [53,54]. The existence of viral antigens causes red blood cells (RBC) to agglutinate [55]. Agglutination could be averted by anti-dengue antibodies, and how effectively this can be curbed can be checked by this test [56]. During primary infection, the HI antibody rose to 1:1640; however, titres of 1:1280 or higher are usual during secondary infection. The test was easy, swift, sensitive, and specific. However, it had shortcomings such as a high chance of cross reaction and the sample also needed to be pre-treated for the removal of non-specific inhibitors of haemagglutination [47]. A study was conducted between the haemagglutination test and IgG ELISA to check whether both these tests can distinguish between a primary and secondary infection. Both these tests could detect primary infections, though when it came to secondary infections, the IgG ELISA test could identify more secondary infections in comparison to the haemagglutination test [57]. The IgG-based ELISA test is simpler to perform than the haemagglutination inhibition test and is more dependable [54].

#### 3.1.5. Plaque Reduction Neutralization Test (PRNT)

This test could determine the exact infection source in patients who are IgM positive by the detection of neutralizing antibodies [58]. It is a gold standard for calculating the level of antibodies for various viral diseases and is generally used in conditions where serological information is required or for the confirmation of a case. It is usually performed in a test tube or microtitre plate [59,60]. A specific antibody holds the capability for neutralization of the virus. The formation of plaque was prevented by virus neutralization. So, theoretically, the inactivation of the virus by the antibody prevents plaque formation [41]. This test is laborious and takes up a lot of time [41].

#### 3.1.6. NS1-Based Tests

The NS1 antigen plays an important role in the replication of the DENV in the host cell. This antigen which is found in the bloodstream of infected patients is taken into account to be a significant biomarker for flavivirus detection in the initial stages [61]. NS1 tests can detect the acute phase of the dengue virus and NS1 lasts longer in blood than viremia [62]. Among the seven NS1-based tests known, four are rapid tests, namely Dengue NS1 Ag STRIP (BioRad, Marnes-la-Coquette, France), Dengue NS1 Detect Rapid Test (InBios International, Seattle, WA, USA), Panbio Dengue Early Rapid (Alere, Waltham, WA, USA) and SD Bioline Dengue NS1 Ag Rapid Test (Abbott, Abbott Park, IL, USA). The other NS1-based tests are Platelia Dengue NS1 Ag ELISA (BioRad), DENV Detect NS1 ELISA (InBios International) and Panbio Dengue Early ELISA (2nd generation (Alere)) [41]. Rapid tests can be performed among limited resources in the absence of proper healthcare facilities and require less than 30 min to perform. Dengue infection is confirmed on the basis of a positive NS1 test, while a negative test does not rule out an infection probability; hence, an IgM-based test must be carried out [58].

### 3.2. Molecular Detection

Nucleic acid tests are molecular tests which need to be carried out in a centralized laboratory and need the expertise of skilled professionals. Also, these tests involve the extraction of RNA or DNA. Such detection methods yield more accurate results [41].

#### 3.2.1. Polymerase Chain Reaction-Based Tests

Among nucleic acid tests, PCR is the most common one and is considered the ideal test for dengue detection at an early stage due to its high sensitivity. In RT-PCR, the steps of the process include viral RNA extraction from different samples, transcription to cDNA, and amplification. Once the RNA is amplified, the fluorescence obtained from it is read by several devices [63]. In this manner, dengue can be detected. The RT-PCR-based platforms which have been developed in recent times can detect all serotypes and even distinguish them from each other [64]. A multiplex RT PCR assay was established a few years back, which determined the serotypes present in the blood and could be employed in a blood transfusion [64]. A single-step RT-PCR-based assay was established where the designed primers could detect and differentiate between the dengue virus and the zika virus, and the yellow fever virus from the chikungunya virus [65]. This method was highly sensitive and exhibited no cross reaction. Conventional PCR, though, needs skilled technicians and laboratory facilities to conduct this method [41].

#### 3.2.2. Isothermal Amplification-Based Tests

This test requires isothermal amplification of genomic DENV RNA, and the procedure requires a certain temperature. Several platforms have utilized isothermal amplification-based methods such as reverse transcriptase loop-mediated isothermal amplification (RT-LAMP), reverse transcription recombinase polymerase amplification (RT-RPA), and nucleic acid sequence-based amplification (NASBA) [63]. The RT-LAMP assay was made as an encouraging detection technique which utilizes a water bath or a heating block for amplification of the virus RNA at a constant temperature (60–65 °C) [66]. In the absence of adequate medical facilities, loop-mediated isothermal amplification (LAMP) is a befitting method for the detection of infectious viruses. The LAMP-based microfluidic device minimizes reagent expenditure, the probability of sample contamination, and saves time [67]. RT-LAMP is fast, inexpensive, sensitive, and specific [41]. NASBA is another common isothermal amplification method. Here RNA is extracted from samples of serum or plasma by silica, then amplified at 41°C in the absence of a thermocycler [68]. The benefit of this method is the single-step isothermal procedure which targets the RNA of DENV samples [69]. The RPA assay can detect up to 10 DNA copies of the target per reaction in 20 min at a constant temperature (37–42 °C) [70]. NASBA and RPA require less cost and can be set up within limited resources for precise dengue detection [41]. The advantages and disadvantages of conventional methods for the detection of DENV is mentioned in Table 3.

## 4. Graphene-Based Methods for the Detection of Dengue Virus

### 4.1. Integrated Tapered Optical Fibre-Based Sensor

Kamil et al. designed a biofunctionalized tapered optical fibre-based sensor with the incorporation of graphene oxide for the detection of DENV II E proteins. GO was deposited at the tapered region and then functionalized using anti-DENV II E protein IgG antibodies. Raman spectroscopy was used to characterize the GO-integrated tapered optical fibre. Further, this sensor was tested with various concentrations of DENV II E proteins [71].

The spectra were compared in advance and after DENV II E protein was administered at 1 µM. The spectrum taken in advance of the reaction for the DENV II E protein resulted in a red shift of 1.023 nm, which could be due to the DENV II E proteins bound to the tapered fibre surface immobilized by anti-DENV II E protein antibodies. This altered the cladding’s refractive index, which resulted in the spectrum being pushed to the right. A blue shift of 0.83 nm was recorded using the negative control with the GO blended biofunctionalized tapered fibre. This implied failure of Avidin binding to the tapered surface and a breakdown of the sensing layer [71]. The sensor showed a sensitivity of 12.77 nm/nM with a detection limit of 1 pM. The sensor exhibited great accuracy, selectivity, and affinity for E protein. It has a dissociation constant;

K_d_ = 1.11 × 10^−9^ M^−1^. The incorporation of GO improved the sensing performance as it doubled the sensitivity value compared to the standard biofunctionalized tapered fibre. Tapered optical fibres in sensors impart compact, flexible and enhanced sensitivity properties at a cost-effective price [71].

### 4.2. QCM-Based Sensor

To detect the serotype of DENV methods such as RT-PCR and for quantification, a plaque-forming unit staining assay can be used [72]. These methods are both costly, time-consuming, and strenuous. To overcome such problems, this method classifies and quantifies the DENV-1 serotype on the basis of molecularly imprinted polymers (MIPs) and mass-sensitive detection. MIPs imitate the role of antibodies or natural receptors by disclosing particular features such as chemical functionality, size, and shape [73]. The synthesis of MIP depends on how the functional monomers and cross-linkers are self organized around a template. Once the polymerization was complete and the template was removed from the polymer, the matrix held particular recognition sites complementary to the template, which permitted selective rebinding of the templates [74]. Incorporating graphene oxide with the polymer matrix improved the end composites’ thermal, mechanical and electrical features [75].

**Figure 2:**
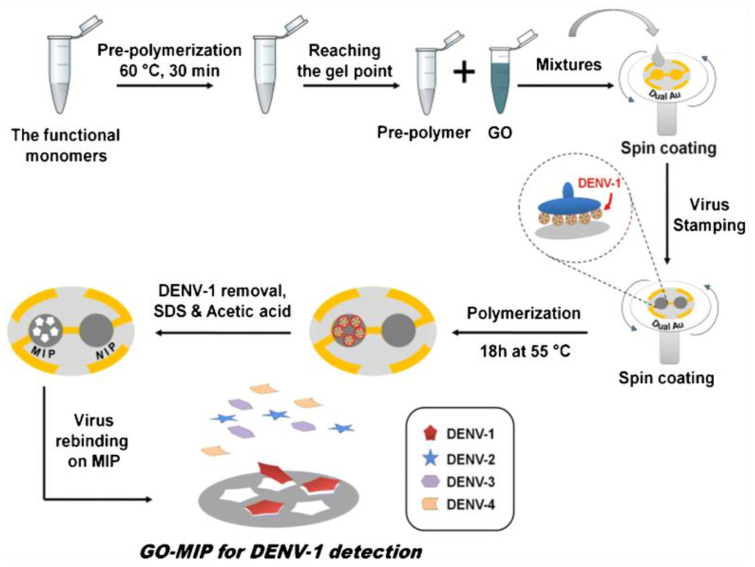
Schematic representation of GO-MIP and corresponding GO-NIP synthesis and their layer fabrication on QCM for detecting DENV-1. Reused with permission from Springer Nature [74].

In this work, an MIP-based sensor recognition material was incorporated with GO which enhanced its sensitivity and detected the DENV-1 (Figure 2). GO was synthesized by Hummer’s method [76]. Polymer composites composed of GO blended with an acrylamide (AAM) methacrylic acid, (MAA) methyl methacrylate, and (MMA) N-vinylpyrrolidone (VP) copolymer were synthesized. The QCM transducers were fabricated, and the DENV-1 imprinted GO polymers were prepared. Then, the polymer composites were characterized by scanning electron microscopy, and QCM measurements were recorded. The zeta potential of the pure polymer was +9.9 ± 0.5 mV, while the zeta potential of the prepared GO sheets was −60.3 ± 2.7 mV. The resultant overall zeta potential of the composite was −11.2 ± 0.2 mV. These polymer composites seemed appropriate for binding the positively charged DENV-1 particle (+42.2 ± 2.8 mV) [74].

Two sets of dual-electrode QCM responses were recorded. Exposure of a device with a DENV-1-MIP and NIP (non-imprinted polymer without GO) to a 10^4^ pfu mL^−1^ DENV-1 standard solution gave the first kind of response, leading to frequency shifts of 595 Hz on the MIP side and −197 Hz on the NIP side, equivalent to the −399-Hz mass effect, proving successful imprinting. The second was from a coated dual composite (GO-MIP and GO-NIP) device. The device was exposed to the standard DENV-1 solution, and signals were obtained for GO-MIP at −1690 Hz and for GO-NIP at −80 Hz, which made a −1610-Hz mass effect overall. GO-MIP makes the net surface charge of thin film shift to negative values. Thus, GO enhances the affinity of MIPs and leads to better sensor response. In comparison to the pure polymers, the response of the composites to the analyte was quicker. The properties of the sensor were investigated in the DENV-1 solution (10^−1^ to 10^4^ pfu mL^−1^). The sensor response was extremely minute at a minimum concentration of 10^−1^ pfu mL^−1^. The limits of detection (LOD) and quantification (LOQ) were calculated and found to be 0.58 and 1.94 pfu mL^−1^, respectively. Thus, the sensor could detect viral concentrations at an early phase of infection with DENV-1. The LOD = 0.60 pfu mL^−1^ and LOQ = 2.0 pfu mL^−1^ for pure MIP were higher than for GO-MIP. So, alteration of surface charge by synthesis of the composite boosts the system’s sensitivity and imprinted polymer composites specifically bind DENV-1 without any intervention. In comparison to standard plaque assay, which requires about seven days, the developed sensor takes 15–20 min to yield the result. It is faster than a cell culture-based method or any nucleic acid amplification method [74].

### 4.3. Electrochemical Paper-Based Analytical Device

An electrochemical biosensor estimates the changes in electrical features such as current, voltage, impedance, and capacitance as a result of redox reactions or biochemical interactions on the surface of the electrode [77]. An electrochemical paper-based analytical device (ePAD) was developed using graphene oxide-silicon dioxide (GO-SiO_2_) nanocomposites to identify DENV 1–4 serotypes. GO-SiO_2_, when used in this device, helped in creating the signal amplification platform. It exhibited wide linearity ranging from 100 pM to 1 μM [78].

The GO-SiO_2_ nanocomposite was synthesized by hydrolyzing the tetraethylorthosilicate (TEOS) [79]. The stencil printing method was used to design the multiplexed paper-based chip [80]. The DNA probes were immobilized in different working areas and the signal response was measured by an electrochemical workstation. GO, and GO-SiO_2_ were characterized by SEM and XRD. The synthesized GO-SiO_2_ nanocomposites were drop-deposited (5 μL) on the circular working area of the ePAD. Then, 3 µL of DENV probe (PDNA) 1–4 serotypes were drop-casted on the four ePAD working areas. The different concentrations of the target (TDNA) of DENV-1, 2, 3 and 4 serotypes (100 pM to 1 μM) were detected by dropping a mixture of TDNA and methylene blue. In order to evaluate the hybridization between the probe and target DNA, cyclic voltammetric measurements were noted. The sensor’s specificity was analyzed by exposure of the PDNA (1–4) to the target samples of complementary and non-complementary serotypes and further measurement by cyclic voltammetry [78].

A calibration curve was required from each DNA serotype for the detection serotype specific DNA in a clinical sample. Therefore, the different concentrations of specific target DNA in the different working regions were examined and electrochemically characterized separately in different working regions. Every assay step was optimized, especially with respect to the PDNA concentrations and the number of incubations of PDNA and TDNA. The cyclic voltammetry results stated that the signal response was reduced with a rise in the concentration of DENV TDNA (100 pM to 100 µM) in all of the active regions. On the other hand, due to the interaction between methylene blue and guanine, the single-stranded DNA resulted in an improved response [78] described in various literature [81].

The GO sheets with the composites exhibited enhanced physical and chemical properties. Such nanocomposites were used in electronic devices, supercapacitors, solar cells, and in environmental, energy, and biomedical applications. The paper-based sensors offer a wide range of advantages such as ease of disposal, mass production, cost effectiveness, tailorability, even distribution of nanoparticles on the surface, the need for minute samples, and convenience of use. This device could be utilized for the detection of each DENV DNA serotype which will be beneficial for the prediction of DHF’s prevalence during secondary infections which was not diagnosed in the initial stages [78].

### 4.4. Loop-Mediated Isothermal PCR

The differential diagnosis of Zika and Dengue was made based on a graphene oxide multiplexed detection method focusing on the viral nucleic acid. This fluorometric detection procedure was combined with a loop-mediated isothermal amplification assay to enhance sensitivity. In this diagnostic process of nucleic acid testing (NAT), virus samples are directly applied to the amplification reaction without any need for the isolation of RNA. This technique aimed to differentiate between the target genome of DENV from the zika virus (ZIKV) and to achieve the detection of DENV serotypes 1 to 4 (S1–S4). This method used a GO-based fluorometric biosensor, which involves sequence-specific peptide nucleic acid (PNA) probes. Also, single-stranded DNA (ssDNA) loops resembling cauliflower virus amplicons were made through a loop-mediated amplification reaction (LAMP) and could be identified later via a sequence-specific fluorescent PNA probe [82].

In virus detection, two methods were used, namely detection between ZIKA and DENV, and multiplexed detection of each DENV-1 to 4 serotypes. The sequences of every target genome for the Zika and Dengue virus were studied in depth to conduct differential diagnosis between them. On the basis of the LAMP assay [83], the single-stranded DNA loop sequences (32–58 nucleotides) of the virus amplicons produced by the amplification reaction were studied to select the target sequence of the PNA–GO sensor. The PNA probes were designed complementary to the midregion in the target loop DNA sequence of virus amplicons. Also, a pandengue virus (pan-DENV) PNA probe was developed, which encloses all the DENV serotypes to study the possibility of differentiating between ZIKV and DENV [82].

In this method, the initial step was modified by curtailing the RNA isolation of the sample, which reduced diagnosis time. The utilization of loop-mediated isothermal amplification (LAMP) in place of PCR reduced the amplification time of RNA with increased sensitivity. The one-step reverse transcription loop-mediated isothermal amplification (one-step RT-LAMP) was achieved by incorporation of the reverse transcription reaction of RNA to DNA to LAMP [82].

The current shortcomings of postreaction analysis were non-specificity and complexity in differentiating between two virus amplicons. Thus, the PNA–GO-based detection technique solves these issues via target–probe hybridization and sequence-specific recognition of virus amplicons. It also succeeds in serotype differentiation by fluorometric multiplexed detection on the basis of the merits of target-specific PNA probes and fluorescence quencher GO. Some difficulties remain with respect to the complexity, cost, and technical problems of the nucleic acid testing field to build a completely equipped detection platform comprising all the steps for point of care [82].

### 4.5. Lateral Flow Immunoassay

In lateral flow immunoassay, a detection label using tapered nitrocellulose (NC) membrane and gold-graphene oxide (Au-rGO) was used to increase sensitivity in the detection of DENV NS1. Colorimetric sensors constitute a detection procedure followed by a change of colour due to specific ligand-target interactions in a solution state or solid support [84]. Gold–graphene oxide (Au-rGO) nanocomposites were prepared by an ex-situ process. The detection antibodies were conjugated to gold-graphene oxide (Au-rGO) nanocomposites by directional conjugation [85]. The nanocomposites were then characterized by a high-resolution transmission electron microscope, Raman spectroscopy, Fourier transform infrared spectroscopy (FTIR) and fluorescence spectroscopy. Fluorescence spectroscopy was used to estimate the number of immobilized antibodies per AuNP. The dengue antigen was presented with more nucleation sites by utilizing Au-rGO nanocomposites exhibiting greater sensitivity to the assay. The gold nanoparticles embedded on rGO sheets were used for labelling, which assisted the immunoassay in displaying increased sensitivity levels. The high surface-to-volume ratio of the rGO sheets prevented the clustering of gold nanoparticles, and thus, the active binding sites increased in number to immobilize the anti-dengue antibodies. The usage of a modified tapered nitrocellulose (NC) membrane levelled up the concentration of antigen-bound nanoparticles at the test line. The signal enhancement led to a much-reduced detection limit of 4.9 ng mL^−1^ [86].

The system possesses multiple properties such as high sensitivity, low runtime (10 min), and ease of manufacturing in comparison to existing reported systems. It could be used in the near future to diagnose closely related viruses such as malaria, zika, etc., which show similar symptoms in patients. A combination of remarkable characteristics of Au-rGO nanocomposites and structural modification increased the level of sensitivity by 11 times in comparison to conventional lateral flow assays [86].

### 4.6. Surface Plasmon Resonance Based Sensors

**Figure 3:**
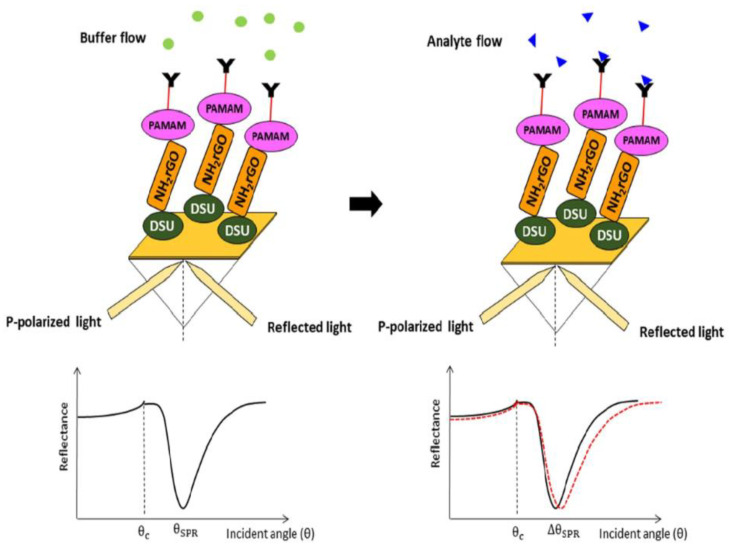
Schematic illustration of the SPR signal before and after the analyte flow. Reused with permission from Springer Nature [88].

A surface plasmon resonance biosensor detects alterations in the target analyte during interaction with a biorecognition constituent on the sensor by surface plasmon waves [77]. An SPR sensor on the basis of self-assembled monolayer reduced graphene oxide-polyamidoamine dendrimer (SAM/NH_2_rGO/PAMAM) thin film for the detection of DENV-2 E-proteins was established. The Au/DSU/NH_2_rGO-PAMAM/IgM sensor film was fabricated. Surface characterizations by X-ray diffraction (XRD) and Fourier-transform infrared spectroscopy (FTIR) confirmed the integration of NH_2_rGO-PAMAM nanoparticles in the prepared sensor films. The SPR measurements were performed on the basis of the Kretschmann configuration [87] by evaporation of Au/DSU/NH_2_rGO-PAMAM/IgM sensor film on the surface of the prism. An optical stage was used for placing the prism, which was driven by a stepper motor and had a resolution of 0.001° (Newport MM 3000), for allowing the incident light from the laser beam (632.8 nm, 5 mW) to pass via the prism to strike the gold layer for the generation of surface plasmon resonance waves at the interface. The SPR response was induced at a particular angle of the incident light during the evanescent wave generation because of an alteration in the refractive index of the medium near the gold surface. The detection system comprised a 100 µL flow cell attachment to the sensor film containing a DENV-2 E-proteins solution (0.08–0.5 pM). The experiments were repeated thrice for every concentration at room temperature. Figure 3 illustrates the proposed Au/DSU/NH_2_rGO-PAMAM/IgM sensor with the administration of DENV-2 E-proteins [88].

**Figure 4:**
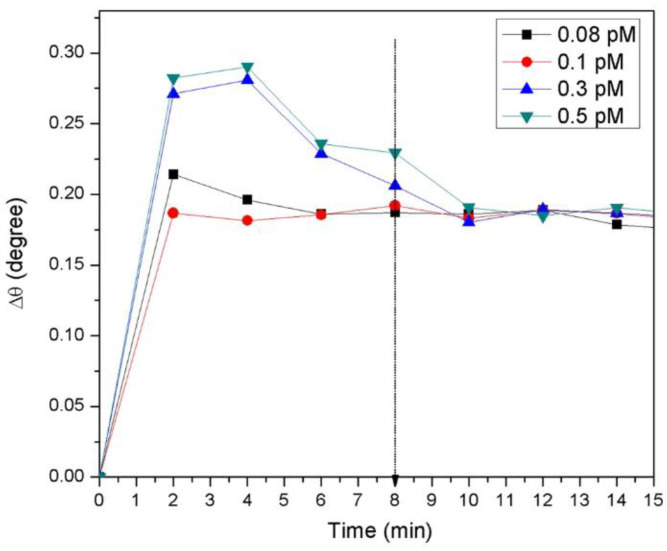
Realtime detection for different concentrations of DENV-2 E-proteins in contact with Au/DSU/NH_2_rGO-PAMAM/IgM sensor film. Reused with permission from Springer Nature [88].

**Figure 5:**
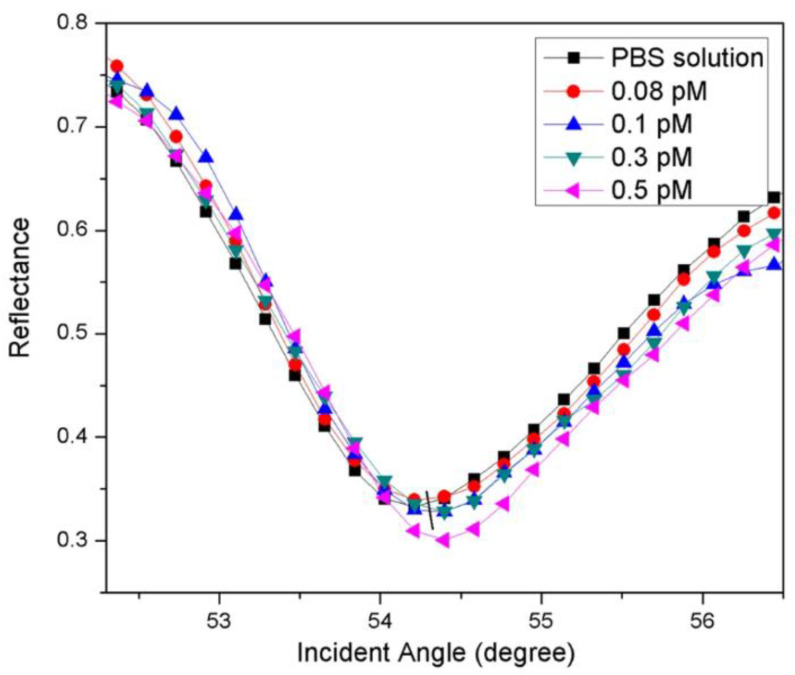
Experimental SPR curves when Au/DSU/NH_2_rGO-PAMAM/IgM sensor film was exposed to 0.08–0.5 pM of DENV-2 E-proteins. Reused with permission from Springer Nature [88].

The SPR realtime measurement of the proposed sensor film for detecting DENV is shown in Figure 4. The different concentrations in the range of 0.08–0.5 pM of DENV-2 E- proteins were administered to the cell to check the viability of the sensor film. The sensor’s response time for detecting high concentrations of DENV-2 E-proteins was 10 min and 6–8 min for the lowest concentration (0.08 pM). This indicated that a rise in concentration led to a spike in the real-time detection of DENV-2 E-proteins. The SPR response (Figure 5) of the sensor film detecting DENV-2 E-proteins was recorded. The resonance angle was 54.2138° in response to the PBS solution. The exposure of the sensor to DENV-2 E- proteins (0.08) pM increased the resonance angle of reflected light to 54.3052°. The angles of resonance from the SPR curves were found to be 54.3137°, 54.3925°, and 54.4004° for 0.1 pM, 0.3 pM, and 0.5 pM concentrations of DENV-2 E-proteins. The resonance angle shift (Δθ) was used to estimate the number of antigens bound to the sensor surface. The resonance angle shifts could be responsible for the alterations in the refractive index of the sensor’s surface [88]. Also, a small angle shift of SPR could be due to a change in the thickness of the sensing layer because the evanescent wave has more depth of penetration [89].

The results revealed that because of different spin speeds and incubation times, the varying sensor layer and concentration yielded more interaction between the sensing layer and the analyte. The sensor exhibited good linearity (R^2^ = 0.92), and DENV-2 E-proteins of concentration as low as 0.08 pM were detected in 8 min [88].

The rGO is advantageous over the GO as it possesses a longer storage duration without clustering, and is stable in organic solvents. A combination of polyadinoamine (PAMAM) dendrimer with rGO paves the way to improve the detection’s sensitivity. PAMAM dendrimers possess multiple benefits in applications related to sensing due to their enhanced binding site on the dendrimer and capability for the transportation of bioactive agents [90], which led to an increase in the number of active sites for binding DENV E- proteins. The thin film-integrated SPR sensor could be developed as a point-of-care device in the future [88].

### 4.7. Impedimetric Immunosensor

In an impedimetric biosensor, a minute sinusoidal voltage at a certain sinusoidal voltage was applied, accompanied by measurement of the resultant current. Next, the current-voltage ratio provides the impedance [13].

**Figure 6:**
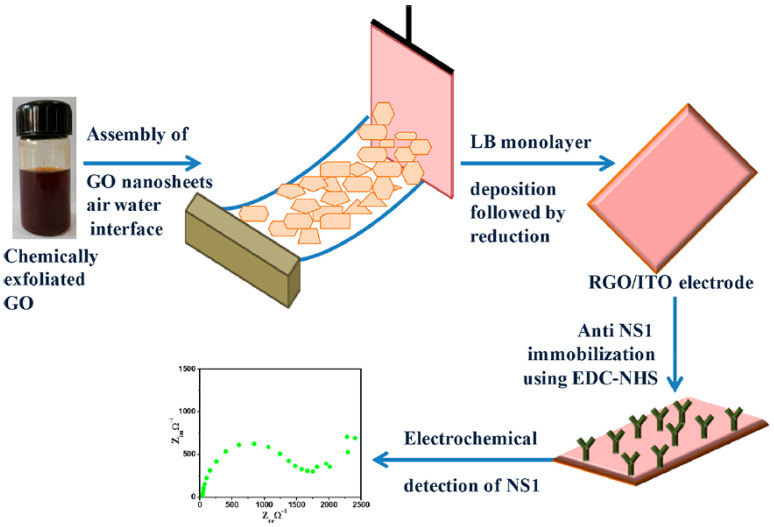
Fabrication of the Bioelectrode and NS1 Detection. Reprinted with permission ref. [92]. Copyright 2021, ACS.

In this work, rGO films were used as transduction materials for detecting NS1 antigens. Electrodes made from partially reduced rGO nanosheets were used for quantifying the NS1 protein, and thus, dengue was detected. GO nanosheets were synthesized using modified Hummer’s and Offeman’s methods [91]. The GO nanosheets were characterized by ultraviolet−visible (UV−vis), and Fourier transform infrared (FT-IR) spectroscopy, X-ray diffraction (XRD) analysis, tunnelling electron microscopy (TEM), atomic force microscopy (AFM), and Raman spectroscopy. The Langmuir-Blodgett (LB) films of the synthesized GO sheets were deposited onto hydrolyzed indium tin oxide (ITO) coated glass substrates in optimized conditions of solvent, pressure, and concentration (Figure 6). Hydrazine vapours reduced these films through the utilization of hydrazine hydrate. The synthesized rGO films were used for the immobilization of anti-NS1 antibodies to fabricate the extremely sensitive impedimetric immunosensor to indicate the quantity of NS1 antigens. The detection limit obtained for standard and spiked samples were 0.069 ng mL^−1^ and 0.081 ng mL^−1^, respectively. Also, a sensitivity of 8.41 and 36.75 Ω per ng mL^−1^ was detected in the range of 10^1^ to 10^7^ ng mL^−1^ [92].

The conditions were optimized for the Langmuir-Blodgett (LB) of rGO films. The fabrication of LB films of chemically exfoliated GO nanosheets was a success, along with the partially reduced rGO. The investigation was performed to assess the structural and morphological properties of the fabricated immunosensor supported strongly for forming GO films under an optimized surface pressure (15 mN m^−1^) on the ITO substrates. The immunosensor has great potential for the diagnosis of dengue and holds the capability to become a point-of-care test. Using this method, large area rGO films could be fabricated easily and inexpensively, which would help in the pilot-scale manufacture of rGO thin films for various applications, including biosensors. The steadiness of the formed film and the sensor’s storage are some of the difficult points which need to be looked into [92].

The different graphene-based sensors for dengue detection are listed in Table 4.

## 5. Conclusions

Dengue infects millions of people each year, with testing being vital to diagnosis and treatment. All tropical and subtropical regions face a serious threat due to this endemic. Early and rapid detection of the virus will better help to manage its spread. It is worth mentioning that no antivirals, drugs, or vaccines are available to protect against the disease burden, and there is a constant search for a remedy. Conventional technologies such as ELISA and PCR are currently being used for their detection. However, these assays are not cost effective, require sophisticated instruments, and lack high sensitivity and specificity. As a result, a rapid, accurate, and early diagnosis is crucial for a successful medical intervention to curb dengue outbreaks and their re-emergence.

Graphene, due to its various advantages, such as its large surface-to-volume ratio, high electrical conductivity, and tailorability, is widely used as a platform for the development of rapid point-of-care diagnostics. Several GO and rGO-based virus detection biosensors have already been explored and reported. The dispersibility of graphene-based nanocomposites could also be utilized as an ink for ink-printed electrodes and paper-based sensors which could widen the application of such materials in biosensors and promote miniaturization. An important focus should be made towards standardization, miniaturization, and multireadout biosensors that could reduce the rate of false positives and negatives. Such properties, in conjugation with enhanced sensitivity, selectivity, and huge production, are significant for the wide application of graphene sensors for the detection of a variety of viruses. There is immense potential in the incorporation of graphene sensors with smartphones that ensures less cost, trustworthiness and an all-around platform for biosensing which could be broadly applied in POC devices. The development of microfluidic biosensing setups, lateral flow assays and smartphone-based biosensing methods will be prominent in the future for the development of biosensors for dengue detection.

Even though biosensors offer better alternatives to traditional methods and give cost-effective, sensitive, swift, miniature and portable platforms, the development of biosensors often faces a lot of challenges. Some of the challenges that should be addressed during the development of biosensors are the optimization of platforms and enhancement in the functioning of wearable biosensors, optimization in the development of ingestible biosensors incorporated with electronic types of equipment for real-time in vivo monitoring, and the development of precise and swift biosensors combined with remote control systems for monitoring the environment. The present review focuses on graphene and its derivatives as an effective diagnostic for detecting the dengue virus. Looking at its immense capabilities, graphene and its derivatives hold a promising future and can be further explored to make it more affordable and sensitive.

## Figures and Tables

**Table 1:** Graphene-based detection for different viruses. (H1N1—haemagglutinin type 1 and neuraminidase type 1 influenza virus, HPV—human papilloma virus, HSV1—herpes simplex virus 1, NoV—norovirus, HCV—hepatitis C virus).

Virus Name	Electrode Material	Method of Detection	Reference
H1N1	Graphene oxide	Electrochemical	[31]
H1N1	Reduced graphene oxide	Electrochemical	[32]
HPV	Reduced graphene oxide	Electrochemical	[33]
HPV	Reduced graphene oxide	Field effect transistor	[34]
HSV1	Reduced graphene oxide	Optical	[35]
NoV	Graphene oxide	Optical	[36]
HCV	Reduced graphene oxide	Optical	[37]

**Table 2:** Conventional methods of dengue detection.

Method of Detection		Reference
Serological tests	IgM-based tests	[31]
	IgG-Based Tests	
	IgM/IgG Ratio Tests	
	Haemagglutination Inhibition Test	
	Plaque reduction neutralization test	
	NS1-based tests	
Molecular detection	PCR-based tests	[31]
	Isothermal Amplification-Based Tests	

**Table 3:** Advantages and disadvantages of conventional methods for the detection of dengue (Reused with permission from Biosensors, MDPI [41]).

Detection Method	Advantages	Disadvantages	Target
Serological	Comparatively fast, easier to execute, less expensive	Expensive device required shows cross reactivity	NS1, IgA, IgG and IgM
PCR	Accurate, early-stage detection, muliplexibility, highly sensitive and specific	Only suitable for high resource available settings, skilled personnel needed, prone to contamination, laborious, and time consuming	RNA
Isothermal	Fast, no need of thermocycler, simpler than PCR, early-stage detection	Less multiplexibility than PCR, prone to primer dimer due to high no. of primers	RNA

**Table 4:** Graphene-based detection of dengue virus (Pfu—Plaque forming unit, mL—mililitre, pM—picomolar, μM—micromolar, ng—nanogram).

Electrode Material	Method of Detection	Limit of Detection	Reference
Graphene oxide	Optical	1 pM	[71]
Graphene oxide	Electrochemical	100 pM to 1 μM	[59]
Graphene oxide	Colorimetric	4.9 ng mL^−1^	[86]
Reduced graphene oxide	Optical	0.08 pM	[88]
Reduced graphene oxide	Electrochemical	10–10^7^ ng mL^−1^ M	[92]

## Data Availability

Not applicable.
